# Tube-side mass transfer for hollow fibre membrane contactors operated in the low Graetz range

**DOI:** 10.1016/j.memsci.2016.09.049

**Published:** 2017-02-01

**Authors:** C.Y. Wang, E. Mercer, F. Kamranvand, L. Williams, A. Kolios, A. Parker, S. Tyrrel, E. Cartmell, E.J. McAdam

**Affiliations:** Cranfield Water Science Institute, Vincent Building, Cranfield University, Bedfordshire MK43 0AL, UK

**Keywords:** Lumen, Graetz-Lévêque, Graetz problem, Low Reynolds number, Entrance region

## Abstract

Transformation of the tube-side mass transfer coefficient derived in hollow fibre membrane contactors (HFMC) of different characteristic length scales (equivalent diameter and fibre length) has been studied when operated in the low Graetz range (*Gz*<10). Within the low *Gz* range, mass transfer is generally described by the Graetz problem (*Sh*=3.67) which assumes that the concentration profile comprises a constant shape over the fibre radius. In this study, it is experimentally evidenced that this assumption over predicts mass transfer within the low Graetz range. Furthermore, within the low *Gz* range (below 2), a proportional relationship between the experimentally determined mass transfer coefficient (*K*_*ov*_) and the Graetz number has been identified. For *Gz* numbers below 2, the experimental *Sh* number approached unity, which suggests that mass transfer is strongly dependent upon diffusion. However, within this diffusion controlled region of mass transfer, tube-side fluid velocity remained important. For *Gz* numbers above 2, *Sh* could be satisfactorily described by extension to the Lévêque solution, which can be ascribed to the constrained growth of the concentration boundary layer adjacent to the fibre wall. Importantly this study demonstrates that whilst mass transfer in the low Graetz range does not explicitly conform to either the Graetz problem or classical Lévêque solution, it is possible to transform the experimentally derived overall mass transfer coefficient (*K*_*ov*_) between characteristic length scales (*d*_*h*_ and *L*). T_*h*_is was corroborated by comparison of the empirical relationship determined in this study (*Sh*=0.36*Gz*) with previously published studies operated in the low *Gz* range. This analysis provides important insight for process design when slow tube-side flows, or low Schmidt numbers (coincident with gases) constrain operation of hollow fibre membrane contactors to the low *Gz* range.

## Highlights

• For the low Graetz range (<2), a proportional relationship between Sh and Gz is found.
• For *Gz*<2, Sh depends on diffusion but fluid velocity remains important.
• For *Gz* numbers above 2, *Sh* satisfactorily described by extension to Lévêque.
• Experimental *K*_*ov*_ can be transformed between length scales (*d*_*h*_ and *L*) at low Gz.

## Nomenclature

A: mass transfer area provided by the membrane (m^2^)
a: specific surface area (m^−1^)
D: diffusivity of water vapour in the gas phase (m^2^ s^−1^)
d_h_: hydraulic diameter (lumen or inner fibre diameter) (m)
k: specific mass transfer coefficient (m s^−1^)
K: overall mass transfer coefficient (m s^−1^)
L: length of hollow fibres (m)
P: membrane permeability (barrer (1×10^−10^ cm^3^ cm^−2^ cm^−1^ s^−1^ cm Hg^−1^))
Q: flow rate (m^3^ s^−1^)
R: gas constant (J K^−1^ mol^−1^)
RH_in_: relative humidity of inlet gas (%)
RH_out_: relative humidity of outlet gas (%)
T_1_: water vapour concentration in the air phase (ppm)
V: water vapour concentration in the air phase (ppm)
$X_{\mathrm{Sat}}^{W}$: water vapour concentration in the air phase (ppm)
$X_{\mathrm{Sat}}^{W}$: saturated water vapour concentration (ppm)

**Greek letters**
ρ: density of nitrogen gas (kg m^−3^)
µ: dynamic viscosity of nitrogen gas (kg m^−1^ s^−1^)
δ: membrane thickness (m)

**Dimensionless numbers**
Gz: Reynold number [$\rho d_{h}v/\mu$] –
Pe: Reynold number [$\rho d_{h}v/\mu$] –
Re: Reynold number [$\rho d_{h}v/\mu$] –
S: dimensionless Raoult's law constant for water –
Sc: Schmidt number [μ/ρD] –
Sh: Sherwood number [$kd_{h}/D$] –

**Subscript**
f: feed
g: gas phase
l: liquid phase
L: liquid phase
s: solvent
ov: overall

**Subscript**
a: air
w: water

## 1 Introduction

Hollow fibre membrane contactor (HFMC) technology has been demonstrated as a mass transfer process for numerous gas-liquid applications including oxygen desorption for industrial scale boilers [14], from ultrapure water [34], for absorption applications [5], [43] and at smaller scale, for blood oxygenation in open heart surgery [9]. Their commercial advantage can be ascribed to the phase separation facilitated by the membrane, which promotes non-dispersive mass transfer between fluids, whilst simultaneously permitting considerable specific surface area to be incorporated into a module, thus providing large volumetric mass-transfer coefficients (*Ka*) to be achieved relative to conventional mass transfer technologies [3].

For industrial scale design of HFMC, an engineer needs to establish the overall separation, given a specific feed concentration, which can be correlated in terms of the average or overall mass transfer coefficient (*K*_*ov*_) [11]. The *K*_*ov*_ can then be used to establish the contactor length (*L*) necessary to achieve a stated treatment objective [32]:

(1) $$L=\left[\frac{V_{0}}{K_{\mathrm{ov}}a}\right]\left\{\left(\frac{\frac{Q_{s}M}{Q_{f}}}{\frac{Q_{s}M}{Q_{f}}-1}\right)\ln\left(\frac{y_{0}-Mx_{0}}{y_{1}-Mx_{1}}\right)\right\}$$

where *V*_0_ is the superficial feed velocity, *a* is the surface area of membrane per volume of bed, *Q* is the solvent (s) and feed (f) flow, M is the partition coefficient, and *x* and *y* are the solute mole fractions in the feed and solvent respectively [32]. The mass transfer coefficient used in (1) can be adapted from the literature but requires correction when the characteristic length employed during determination of *K*_*ov*_ is different to that proposed in the design. In the case of tube-side flow, the characteristic length is the inner tube diameter (*d*_*h*_) and translation of *K*_*ov*_ between length scales can be achieved using the dimensionless Sherwood number (*Sh*):

(2) $$\mathrm{Sh}=\frac{K_{\mathrm{ov}}d_{h}}{D}$$

where *D* is mass diffusivity. As the Sherwood number represents the ratio of total rate of mass transfer to the rate of diffusive mass transport, it is implicitly governed by the fluid dynamics employed. Provided that tube-side flow is characteristically laminar, the Graetz number ($\mathrm{Gz}=\mathrm{ReSc}d_{h}/L$) can be used to describe fluid behaviour through [28]:

(3) Sh=aRebScc(dhL)e

where *Re* and *Sc* are Reynolds (Re=ρdV/μ) and Schmidt numbers (Sc=μ/ρD) respectively. Importantly, inclusion of the $\left(d_{h}/L\right)$ term emphasises that *K*_*ov*_ is dependent on both the tube-side diameter and fibre length.

For gas-liquid applications with gas flowing on the tube-side, *K*_*ov*_ represents contributions from the membrane (*k*_*m*_), liquid phase (*k*_*l*_) and gas phase (*k*_*g*_) [17]. However, the overall resistance to mass transfer ($1/K_{\mathrm{ov}}$) is often dominated by one or more phases. For example, during an air stripping study with air flow on the tube-side and water surrounding the external periphery of the fibre, Mahmud et al. [22] suggested that the liquid phase resistance to mass transfer was negligible, thus the residual resistance was accounted for by both the membrane resistance and air-phase resistance developing on the tube-side. For membranes with thin walls, where solutes exhibit high permeability [28] or for applications constrained to low flows, the membrane resistance can be considered negligible, and the concentration boundary layer developing in the fluid adjacent to the membrane wall, will generally dominate mass transfer resistance [10], [28].

Numerous authors have demonstrated that the Lévêque solution can be usefully applied to estimate the tube side mass transfer coefficient (Table 1) [6], [24]:

(4) $$\mathrm{Sh}=1.6151{\mathrm{Gz}}^{1/3}$$

This solution proposes that the concentration boundary layer is limited to a thin layer adjacent to the fibre wall, and so is only valid for relatively high fluid velocities (*Gz* generally exceeding 20) through short fibres operating in laminar flow [16]. However, for applications where only low driving pressures are available [2], or where pressure drop is constrained to promote energy conservation [6], often only low Graetz numbers (below Gz of 20) can be achieved. Operation in the low Graetz range is compounded when the tube-side fluid is gaseous, as the Schmidt number reduces to around 1 from a value of around 1000 for liquids. For small values of *Gz*, that are coincident with slow flows, low *Sc* and long tubes, Graetz proposed an approximate solution for the differential equation driven from the continuity equation, by which the average and local Sherwood number can be obtained for conditions below *Gz* of 10 [16]:

(5) Sh=3.67

This solution assumes that the concentration profile is completely developed, indicating a constant shape over the radius [18]. Through curve fitting of (4) and (5), Kreulen et al. [18] proposed the following solution to describe the *Gz* range:

(6) $$\mathrm{Sh}=\sqrt{3.67^{3}+1.62^{3}\mathrm{Gz}}$$

Analytical comparison of (4) and (6) with the available experimentally derived Sherwood data from the literature evidences several important points [23], [24], [30], [41]:

(i) the Lévêque solution provides general agreement for Gz numbers exceeding 20, and apparently satisfactory description of experimental *Sh* data above *Gz* around 5. The solution can therefore be used to provide an estimation of the mass transfer coefficient, when *Gz* exceeds 5;
(ii) below *Gz* of around 5, experimental *Sh* data tends away from the available analytical solutions toward unity as the *Gz* number is reduced; and
(iii) both the Lévêque and Graetz solutions grossly overestimate experimental *Sh* data reported within the low Graetz range (*Gz*<5).

Such disparity has been previously reported in packed bed reactors operated at low Péclet numbers [19]. Numerous experimental membrane contactor studies operated below a *Gz* of 10, have similarly evidenced such a phenomenon [7], [15], [24], [31], [41]. However, there is only limited knowledge of how the Sherwood number changes within the low Graetz range. Specifically, whether mass transfer in HFMC achieves a constant value or decreases as the Graetz number is reduced is currently unclear. Furthermore, as the existing analytical solutions do not appear to adequately describe the experimentally determined *Sh* within the low Graetz range, there is no existing knowledge as to whether the rate of mass transfer can be estimated through transformation of the Sherwood number using the characteristic length scales, *L* and d_h_. The aim of this study is therefore to investigate tube-side mass transfer in hollow fibre membranes operated within the low Graetz range. Specific objectives are to: (i) identify whether the Sherwood number approaches a constant value within the low Graetz range; (ii) determine whether Sherwood analysis can describe mass transfer across a broad range of tube diameters within the low Graetz range; (iii) determine the impact of tube length on Sherwood analysis conducted in the low Graetz range; and (iv) compare this discrete Sherwood analysis conducted on single hollow-fibre membranes to the literature data produced from multi-fibre modules to determine whether analogous behaviour is observed.

## 2 Materials and methods

### 2.1 Experimental setup

**Fig. 1:**
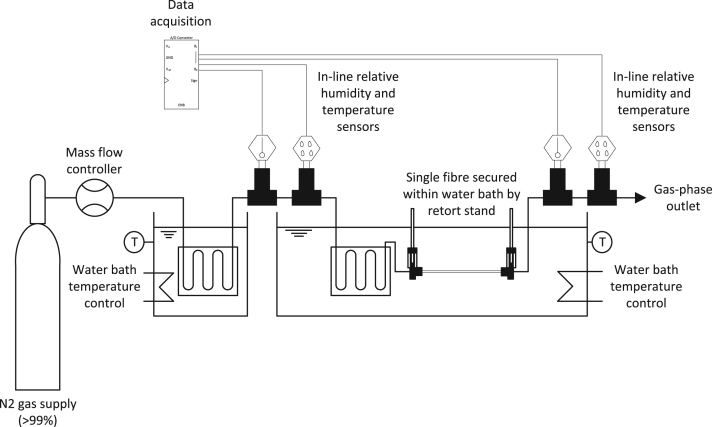
Schematic of single fibre experimentation.

To investigate the translatability of mass transfer data between characteristic length scales (*d*_*h*_ and *L*), hollow-fibre membranes with seven different inner fibre diameters and four different lengths were specified, all of which comprised identical wall thickness and membrane material (polydimethylsiloxane, PDMS). Wickramasinghe et al. [41] observed a decline in experimental *Sh* data with a reduction in Gz number, and attributed the behaviour to non-uniform flow caused by polydispersity in hollow fibre diameter between fibres in the bundle. In this study, mass transfer analysis was undertaken on single fibres to obviate such flow distribution effects, characteristic of multi-fibre bundles. Each PDMS membrane fibre was potted at both ends with individual end caps (of polypropylene construction) measuring 42 mm in length. This is greater than estimated entrance lengths which were below 36 mm (and were predominantly below 16 mm), indicating that flow was fully developed upon transition into the active length of the fibre. Each test comprised of a single-fibre which was held in place using a dual clamp retort stand (Fig. 1). The fibres were supported in the dual clamp retort stand; the clamps were applied to the potted fibre ends. Both clamps were sited vertically downwards, with the fibre running parallel to the retort bar. The clamps were positioned to ensure the fibre was taut thereby producing a ‘straight’ (i.e. with respect to the retort bar) orientation without inducing unnecessary stretching. The fibre was immersed in a water bath to provide an excess of water to the fibre periphery. Whilst the PDMS fibre construction was of stable soft ‘rubbery’ material, once both fibre ends were clamped to ensure a solid fixing and the fibre installed within the water bath, no fibre movement was visually evident. Dry nitrogen gas (99% purity) was supplied from a cylinder (BOC Ltd., Manchester, England) to the lumen side of the hollow fibre. Nitrogen flow rate was regulated using mass flow controllers with a range of either 0.5–50 ml min^−1^ (Cole-Parmer Instrument Co. Ltd., London, UK) or 0.005–1 l min^−1^ (RM&C Ltd., Sheffield, UK), dependent upon which membrane fibre diameter was under investigation. Pressure drop was highest in the smallest diameter PDMS fibre. However, in all experiments, internal fibre pressure drop was below 0.74 bar(g). Throughout the trials there were no visual observations of fibre deformation following changes to hydrodynamic conditions. The mass flow controllers had an accuracy of ±0.8% of reading or ±0.2% full-scale. Volumetric flow rates downstream of the membrane were verified using by milli-gas counter (MGC, Ritter, North Marston, UK). The nitrogen gas supply passed through two columns in series, both of which were immersed in water baths (X12, Thermofisher, Suffolk, UK) to ensure that isothermal conditions were achieved (∆T=0) between liquid and gas phases. The gas and liquid phases were kept at a constant temperature of 21.5 °C, and the temperature difference between gas phase inlet and outlet was consistently below 0.25 °C. Non-immersed pipework was insulated using proprietary pre-insulated stainless steel pipework (Swagelok, London, UK). Temperature and humidity probes (Vaisala Ltd., Birmingham, UK) were sited upstream and downstream of the immersed fibre, and were supplied calibrated with a manufacturer stated range and accuracy of 0–100% and ±1% respectively. Temperature and relative humidity data were recorded using a 12-bit datalogger with a resolution of 15 s between readings (Picolog 1000 series, Pico Technology Ltd., St. Neots, UK).

To verify humidity sensor response, two nitrogen gas streams (dry nitrogen gas and a saturated nitrogen gas) were partially mixed to provide gases with 20%, 40%, 60%, and 80% humidity. The resultant humidity data was within 2% of the expected values. Prior to experimentation, a non-permeable pipe was installed in place of the membrane and a difference in relative humidity between the inlet and outlet of less than 1% recorded which evidenced the rig was gas-tight. As the membrane fibre was immersed in water, the resistance associated with the liquid phase ($1/k_{L}^{W}S$) can be neglected (see Appendix D for experimental verification) and only the resistances associated with the membrane and gas phase need be considered [22]:

(7) $$\frac{1}{K_{L}}=\frac{1}{K_{\mathrm{OV}}^{W}S}=\frac{1}{k_{m}^{W}S}+\frac{1}{k_{g}^{W}S}$$

The change in water vapour concentration in the air stream in the axial direction on the lumen side of a single hollow fibre under steady state conditions can be given by [22]:

(8) $$\frac{v^{a}dX^{w}}{\mathrm{dZ}}=K_{\mathrm{ov}}^{W}a\left(X_{\mathrm{sat}}^{W}-X^{W}\right)$$

on the assumption of the boundary conditions $X^{W}=0\;\mathrm{at}\;z=0\;\mathrm{and}{\;X}^{W}=X_{L}^{W}\;\mathrm{at}\;z=L$, then:

(9) $$X_{L}^{W}=X_{\mathrm{sat}}^{W}\left(1-e^{-\left(K_{\mathrm{OV}}^{W}\mathrm{aL}/v^{a}\right)}\right)$$

from which the overall mass transfer coefficient can be determined. Whilst the Lévêque solution can provide description of the tube-side mass transfer coefficient close to the entrance region of the lumen (which is generally regarded as valid for Gz exceeding 20), Newman [25] offered an extension to the Lévêque solution for small values of L, which offers potential explanation for some of the lower Gz range:

(10) $$\mathrm{Sh}=1.6151{\left(\frac{\mathrm{ReSc}}{L/d_{h}}\right)}^{1/3}-1.2-0.28057{\left(\frac{L/d_{h}}{\mathrm{ReSc}}\right)}^{\frac{1}{3}}+\ldots$$

For the full expansion series, refer to Appendix B. Before each experiment, dry nitrogen gas with a flow rate of 1 l min^−1^ was used to dry the gas rig for at least 60 min before experiments began. In all experiments, the inlet relative humidity was below 3%. Based on experimental data, prediction intervals were calculated using:

(11) Gz=Sht0.95−+1+1n+(Gz−Gzm)2SSxx

where $t_{0.95}$ is the t-distribution fixed to 95% confidence, n is the number of observations, SE is standard error, ${\mathrm{SS}}_{\mathrm{xx}}$ is the sum of squares of deviations from the sample mean, ${\mathrm{Gz}}_{m}$ is the average Graetz number. Each hollow fibre membrane experiment (both for variations in fibre length and for fibre inner diameter) was undertaken in triplicate. The triplicate comprised of testing three independent fibres with identical geometry, and the average of the three experiments used for mass transfer analysis (Appendix A).

### 2.2 Membrane material

The polydimethylsiloxane (PDMS) hollow fibre membranes were procured from a commercial supplier and comprised of lumen diameters: 100, 300, 800, 1300, 1800, 2800 and 4800 µm. The stated wall thickness was 100 µm. For comparison of lumen diameter, each fibre was prepared at a length of 0.2 m. To determine the impact of fibre length, three lumen diameters were selected (300, 1300 and 4800 µm) and prepared at four lengths: 0.1, 0.2, 0.4, and 0.8 m. Prior to experimentation, both fibre wall thickness and lumen diameter were assessed using environmental scanning electron microscopy (eSEM) at an acceleration voltage of 20 kV (XL30, FEI, Oregon, US) for the fibres with smaller lumen diameters (100–1800 µm) or optical microscopy for the larger lumen diameters (2800 and 4800 µm). Fibre samples for SEM were first coated with gold–palladium (Au–Pd) using a cool sputtering SEM coating unit (E5100, Polaron Equipment/Quorum Technologies Ltd., Lewes, UK). Measurements for both lumen (inner tube diameter) and wall thickness were undertaken until the standard error approached a constant value (Appendix A). Data on fibre lumen diameter and wall thickness were transformed into a size distribution using a log-normal distribution function [20].

## 3 Results

### 3.1 Membrane characterisation

**Fig. 2:**
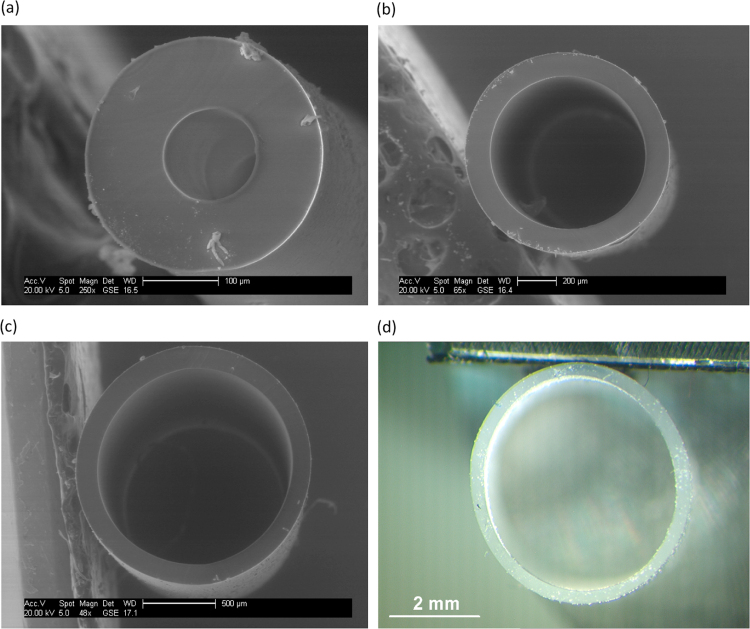
Illustrative environmental scanning electron microscope (ESEM, a–c) and optical microscope images (d) of the hollow-fibre membranes used in this study with lumen diameters of: (a) 100 µm; (b) 800 µm; (c) 1300 µm; and (d) 4800 µm. All fibres comprised a wall thickness of around 120 µm.

A range of seven hollow fibre membrane lumen diameters were selected: 100, 300, 800, 1300, 1800, 2800 and 4800 µm (for illustration, see Fig. 2) to provide a broad data range and to ensure reasonable coverage of the lumen diameters ordinarily selected in the literature [17], [24]. Accuracy of the specified inner diameter (lumen diameter) for each hollow fibre was ascertained using environmental scanning electron microscopy (ESEM) and optical microscopy (Fig. 2, Appendix A). The difference between the stated diameter and the measured diameter were within 6% with the exception of the smallest fibre (100 µm, Table 2). Fibre wall thickness was also measured for each lumen diameter, recording an overall average of 118.5 µm with a standard deviation of 10.8% across all seven fibre samples.

### 3.2 The impact of lumen diameter on mass transfer within the low Graetz range

**Fig. 3:**
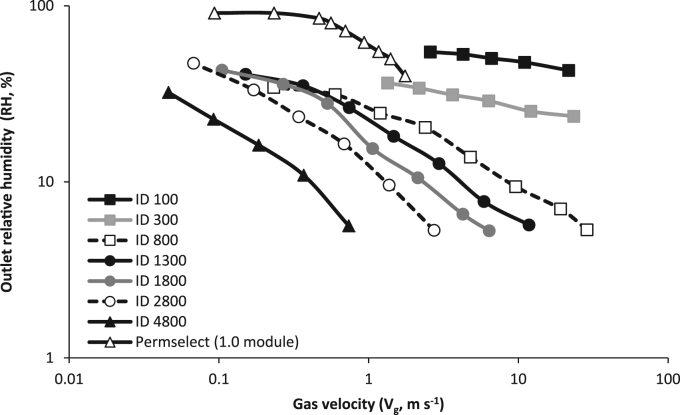
Outlet relative humidity (RH, %) measured in the gas phase at the outlet of each of the hollow-fibre membrane diameters studied (100–4800 µm). Fixed fibre length, 0.2 m.

Outlet relative humidity (RH) was measured directly within the gas phase downstream of the membrane to provide quantification of the overall mass transfer coefficient (Fig. 3). For each lumen diameter, the outlet RH was highest at the lowest gas velocity (*V*_*g*_) tested and declined upon increasing *V*_*g*_. The highest outlet RH was achieved using the fibre with the smallest diameter (100 µm). Outlet RH for fibres with internal diameters of 100 and 300 µm was reasonably insensitive to an increase in *V*_*g*_ when compared to larger diameter fibres operated within the same velocity range. Transformation of the RH data into the overall mass transfer coefficient (*K*_*ov*_) demonstrated that *K*_*ov*_ increased with an increase in gas flow rate (Q_G_).

**Fig. 4:**
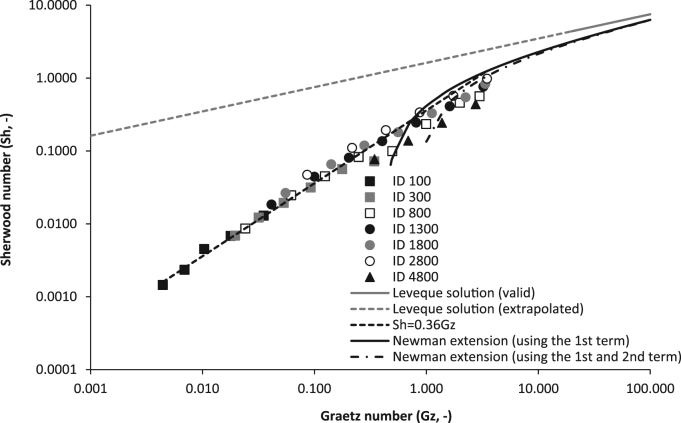
Comparison of mass transfer data for the complete range of lumen diameters studied (100–4800 µm). Fixed fibre length, 0.2 m. Data transformed through Sherwood Graetz analysis to account for variation in hydraulic diameter (d_h_, lumen diameter).

**Fig. 5:**
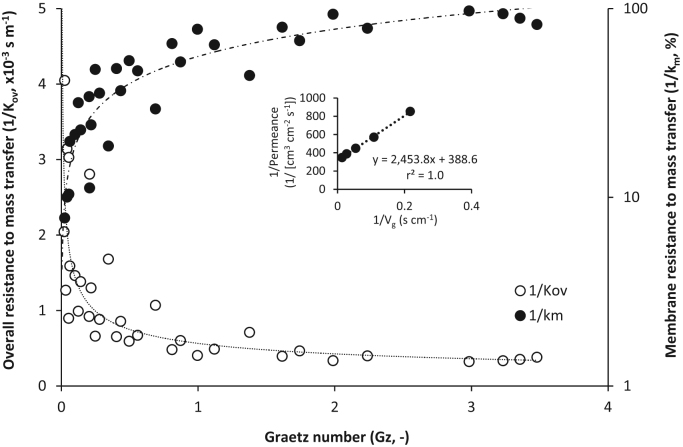
Overall resistance to mass transfer identified for each fibre diameter tested. Inset an example Wilson plot (800 µm fibre ID) used to derive the membrane resistance (1/k_m_, s m^−1^) for each fibre diameter (100–4800 µm ID), which is plotted as a proportion of the total resistance (%).

The Sherwood number was used to normalise the overall mass transfer coefficient for the inner diameter (Eq. (2), Fig. 4) and the Graetz number also applied to enable normalisation of the fluid flow characteristics by inclusion of the hydraulic diameter and fibre length. The full dataset from seven fibres was observed to collapse into a linear slope that was characterised by a gradient of *Sh*=0.36Gz (black dashed line, Fig. 4). Sherwood numbers of between 1×10^−3^ and 1 were obtained from transformation of the overall mass transfer coefficient, and was markedly below that estimated through extrapolation of the Lévêque solution. Membrane resistance was determined for each fibre through Wilson plot analysis with the inverse gas velocity raised to the power of one ($1/V_{g}$, Fig. 5, inset). Resistance in series analysis indicated that for *Gz* of below around 2, resistance to mass transfer was primarily associated with the gas phase on the tube-side. However, once *Gz* exceeded around 2, membrane resistance (1/*k*_*m*_) dominated mass transfer. The membrane resistance (1/k_m_) estimated at the intercept of the Wilson plot can be considered an estimate of the reciprocal of the permeance (or, δ/P) [17], [26]. In this study, the estimated permeance was around 2550 GPU (1 GPU=10^−6^ cm^3^ (STP) cm^−2^ s^−1^ cm Hg^−1^) which compares to a permeance of 575 GPU estimated from literature permeability data ([35] after [21]). Both Bennett et al. [4] and Oliveira et al. [28] reported their experimentally determined PDMS permeability to be considerably higher (up to two orders of magnitude) than the corresponding literature permeability value. The authors explained the difference to arise from the use of different types of silicone membranes. The molecular properties of PDMS including the polymer backbone chemistry, crosslink density and fillers, which are used for promotion of mechanical integrity as well to introduce other unique properties into the polymer, are known to strongly affect water vapour permeability [38]. To illustrate, Van Reeth and Wilson [37] identified over two orders of magnitude in water vapour transmission rate through silicones due to changes in silicon chain length and substitution of the PDMS substrate. In this study, a water flux of around 12 g m^−2^ h^−1^ was determined in the Gz region where 1/k_m_ controlled mass transfer and is equivalent to estimated fluxes from several studies including that of a highly crosslinked PDMS film of equivalent thickness (100 µm; [27], [40]).

### 3.3 The impact of fibre length on mass transfer within the low Graetz range

**Fig. 6:**
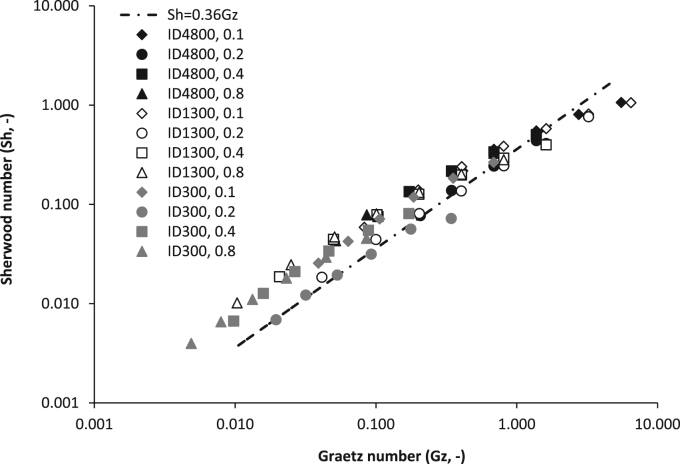
Comparison of mass transfer data through a range of fibre lengths (0.1–0.8 m) for a selected number of fibres (300, 1300 and 4800 µm). Data transformed through Sherwood Graetz analysis to account for variation in fibre length and hydraulic diameter (d_h_, lumen diameter).

**Fig. 7:**
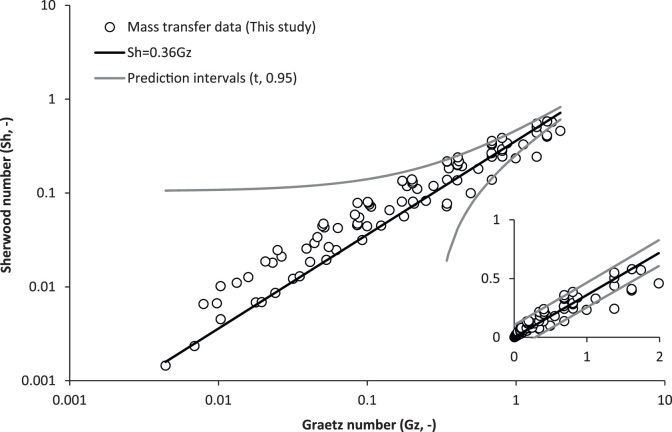
Empirical Sherwood correlation (*Sh*=0.36*Gz*) derived from fibre diameter data is compared to both fibre diameter and fibre length data (*n*=102). Statistical analysis evidenced 93% of data was within prediction intervals (t=0.95), within Gz range 4×10^−3^ and 2. Inset: Linearised dataset.

To determine whether mass transfer data can be extrapolated between membrane fibres of different geometries within the low Graetz range, variations in fibre length (0.1, 0.2, 0.4 and 0.8 m) were also trialled for three membranes of different internal diameter (300, 1300 and 4800 µm) (Fig. 6). Only mass transfer data from the region dominated by gas phase resistance was transformed into the Sherwood analysis (with use of the gas phase diffusion coefficient). Similar to the lumen diameter data, Sherwood data arising from fibres of different lengths collapsed into a linear trend coinciding with Graetz numbers between 5×10^−3^ and 1.7. The preliminary proportionate trend identified between *Sh* and *Gz* (*Sh*=0.36Gz) broadly described the data, and particularly for data below *Gz* of 2. To test this assertion, all Sherwood data produced below *Gz* of 2 were compared to the prediction when fitted with prediction intervals at *t*_*0.95*_ (Fig. 7). For the total dataset (*n*=102), 93% of data was found to lie within the prediction intervals.

### 3.4 Comparison of experimental data with literature data from within the low Graetz range

**Fig. 8:**
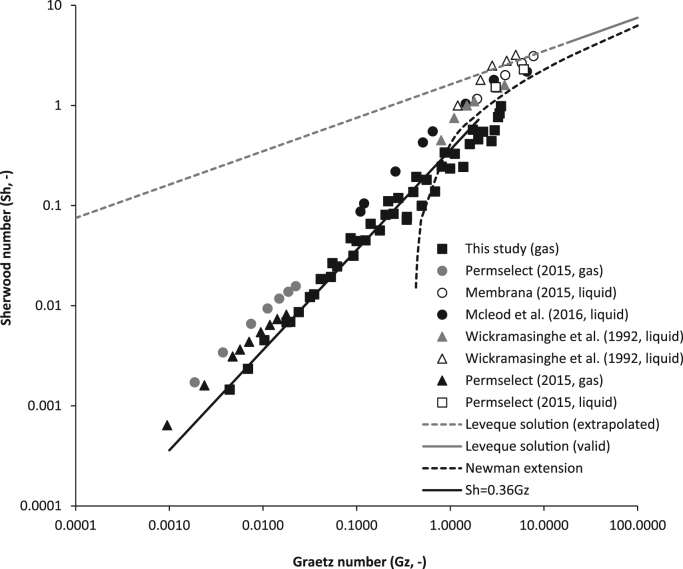
Comparison of experimental mass transfer data from this study with that collated from the literature. Sherwood data extrapolated from [[41] (microporous); [24] (microporous)] and calculated from experimental data for [[30] (nonporous); [23] (microporous)]. Mass transfer data from this study and [30] are provided for the gas phase, whereas [23], [24], [30], [41] are provided for the liquid phase.

Sherwood analysis of the experimentally determined mass transfer coefficient (which was determined in this study using single fibres) was compared to data from the literature (Fig. 8). The published data were either extrapolated from already completed Sherwood analyses [24], [41], or were estimated through calculation of the overall mass transfer coefficient following transformation of reported overall conversion data [23], [30] (Appendix A). The experimentally determined *Sh* data collated comprises of studies investigating tube-side mass transfer for both gases [30] and liquids [23], [24], [41]. The proportional relationship between *Sh* and *Gz* identified in this study during gas phase controlled mass transfer, also provided adequate description of literature data within the same Gz region (below 2). Whereas extension of the Lévêque solution proposed by Newman [25] appears to provide adequate description of the literature data when Gz exceeds 2, which in this study was coincident with the onset of membrane controlled mass transfer.

## 4 Discussion

In this study, a proportionate relationship between Sherwood number and Graetz number has been identified for the region of gas phase controlled mass transfer (below around a Gz of 2) in tube side operation through systematic investigation of the experimentally determined overall mass transfer coefficient within the low Graetz range. Gabelman et al. [15] developed an analytical model using the local mass transfer coefficient to estimate *Sh* across the *Gz* range, and found *Sh* reached a near constant value when *Gz* was reduced below 10 (*Sh*=3.66), which is in close agreement with that of the Graetz problem applied for constant wall concentration (Eq. (5)). However, when compared to experimentally determined *Sh* data, both models were found to overestimate *Sh* within the low *Gz* range (Gz<10). Sieder and Tate [33] studied tube-side heat transfer within the laminar regime, where the experimentally determined heat transfer coefficient was proportional to the fluid flow rate to the 1/3rd power for *Re* exceeding 10, but for *Re* below 7, the heat transfer coefficient was proportional to *Re* to the first power (Table 1) [33]. The results of these authors support the findings in this study, that the experimentally determined overall mass transfer coefficient is not independent of the Graetz number within the low Graetz range, as proposed by (Eq. (5)). Cooney and Poufos [7] operated a HFMC for liquid-liquid extraction at *Re* below 10 and similarly indicated that *Sh* was proportional to *Re* to the first power, which further corroborates the findings of this study. Due to the linear dependence identified between *K*_*ov*_ and *V*_*g*_ within the low *Gz* range in this study, membrane resistance was determined using the Wilson method plotted against 1/V rather than 1/V^0.33^ [7]. Below a *Gz* of 2, the gas phase boundary layer (1/k_g_) was observed to govern the resistance to mass transfer, and a linear dependence between *Sh* and *Gz* was identified. As Gz reduced to below 2, the experimental *Sh* number approached unity, which indicates that mass transfer is then strongly dependent upon diffusion (or free convection) rather than forced convection. However, this study has identified that for this diffusion controlled region of mass transfer, tube-side fluid velocity remains important within the low *Gz* range. Importantly, this study has also demonstrated that within this low *Gz* range, it is possible to translate the experimentally determined *K*_*ov*_ between characteristic length scales (*d*_*h*_ and *L*) and has evidenced that an empirical relationship derived from experimental *Sh* data provides a good fit to data derived within the gas-phase controlled region of mass transfer, with prediction intervals set to t_0.95_ (Fig. 7).

An increase in gas-side (tube-side) humidity resulted from a reduction in gas velocity. This is analogous to previous research on PDMS membranes for air-humidification [30] which indicates that a longer gas-phase residence time in the lumen is important if saturation is to be achieved. To illustrate, the authors achieved around 50% outlet RH at V_g_ 1.4 m s^−1^ (Fig. 3) which compares to a 36% outlet RH at V_g_ 1.33 m s^−1^ using a 300 µm lumen diameter in this study; the higher RH achieved at similar V_g_ can be accounted for by the thinner wall construction and narrower lumen diameter (45 µm and 190 µm respectively). However, by reducing V_g_ below 0.2 m s^−1^, the authors achieved around 90% outlet RH, which is almost 10 times lower than the V_g_ used in this study. Lower tube-side outlet RH was also determined for larger lumen diameter operated at identical V_g_ (and hence identical residence time) (Fig. 3). This could, in part, be compensated for by extending fibre length. For example, for the 4800 µm fibre operated at Re 14, outlet RH increased from 13.5% at 0.1 m fibre length to 31.9% (0.4 m) and 54.9% at 0.8 m. However, the non-linear increase in RH with fibre length indicates hindrance to mass transfer due to the increase in gas-side water vapour concentration which reduces the driving force for mass transfer [17]. Importantly, the governing role of both residence time and lumen diameter on controlling tube-side gas-phase saturation that has been identified in this study, are evidence that the permeability of the membrane used constrains mass transfer. Several previous studies of water vapour mass transport across the membrane barrier have employed either microporous membranes [22] or thin film composites (TFC) [29], [39], [42] and have demonstrated that saturation is achievable. Whilst the polymer of the TFCs employed present similarly constrained permeabilities, their thin wall construction (around 1 µm) enables tremendously high permeances to be realised (Table A2). This is around an order of magnitude higher than the membrane permeance in this study (Table A2), which substantially increases the probability to achieve saturation of the gas phase within the equivalent residence times employed.

For *Gz* above around 2, the resistance to mass transfer was predominantly associated with the membrane (1/k_m_). Within this *Gz* range, *K*_*ov*_ could be satisfactorily described by extension to the Lévêque solution proposed by Newman [25]. This behaviour can be accounted for by the increasing governance of the PDMS material permeability on mass transport which will constrain growth of the concentration boundary layer adjacent to the fibre wall. Use of Newman's extension with only the first expansion term provided good description of *Sh* data between the valid range of Lévêque's solution (Eq. (4)) and a *Gz* of around 2, below which the estimated *Sh* number quickly tended to zero. Frank [13] as cited by Prasad and Sirkar [31] also identified a good fit between Newman's extension and mass transfer data obtained from a short length of single micro-porous hydrophobic hollow-fibre, although the *Gz* range over which the solution was valid, was not specified. As *Gz* was continually increased above 2, the relative membrane resistance was more varied. These higher *Gz* values were achieved with the larger fibre inner diameters (e.g. 4800 µm) which undergo slight deformation (see Fig. 2) as the mechanical strength provided by the thin symmetric wall (around 100 µm thickness) is less able to maintain the circularity of the lumen at this characteristic scale. This is analogous to the observations of Wickramasinghe et al. [41] who suggested polydispersity in inner fibre diameter to account for lower than expected *K*_*ov*_ at low flows.

Within this study, the experimental *Sh* number, which is based on an averaged inlet and outlet concentration driving force across the fibre, was observed to decrease with *Gz* (Fig. 4). This is in contrast to the *local Sh* number within the low Graetz range, often calculated with reference to the governing one-dimensional convective diffusive equation (Eq. (5)) [11], which seems to reach a constant value as *Gz* decreases below 10 [11]. For this case, it is assumed that the concentration profile has a constant shape over the radius [18]. Prasad and Sirkar [31] suggested that the difference between the theoretical and experimental *Sh* data was because the boundary condition of constant wall concentration applied at a *Gz* below 10 is not strictly valid. This assertion is supported by the work of Acrivos and Taylor [1], who analysed a single reactive sphere with the particle Péclet number approaching zero, and found that near the surface of the sphere, diffusion controlled mass transfer, but at a distance from the surface, convection remained important. Through their study of mass transfer in packed beds, Fedkiw and Newman [11] presented compelling evidence that whilst at large Gz, the distinction between the *Sh* values derived from local and averaged mass transfer coefficients was negligible, but as Gz→0, the measured *K*_*OV*_ was below that of the local coefficient *k*_*f*_. The authors accounted for the difference by the influence of axial dispersion which became significant within the low *Gz* range. Whilst both *averaged* and *local* coefficients are defined quantities, the work of Fedkiw and Newman [11] corroborates the findings in this study, that the experimentally accessible, and design useful *K*_*ov*_, which provides description of the average mass-transfer coefficient across the fibre, has been shown to decrease proportionately with a decrease in *Gz* within the low Graetz range. Several authors have postulated that the lower than expected *Sh* data identified within the low Gz range, was a result of non-uniform flow caused by polydispersity in tube diameter in multi-fibre HFMC modules [15]. Experimental data from this study together with the empirical correlation (*Sh*=0.36Gz) developed within gas-phase controlled conditions (below Gz of around 2), as well as Newman's extension which was evidenced to provide description of the ‘upper’ range of *Gz* values studied, were compared to mass transfer data published with multi-fibre HFMC modules operated within the low *Gz* range (several cases were provided through transformation of raw data to the average *K*_*ov*_, Table A1) (Fig. 8). Whilst some data scatter is evident, particularly in the ‘upper’ range of the Gz numbers studied which could be indicative of dispersion effects, the general trend observed in this study can be seen to satisfactorily describe experimental Sherwood data reported from published studies using multi-fibre modules, and is independent of whether the tube-side fluid is liquid or gas. Therefore whilst we agree that fibre diameter polydispersity will influence mass transfer, it is asserted that the overestimation of experimental *Sh* found with existing models can be accounted for by the important role that convection continues to play within the low *Gz* range.

## 5 Conclusions

Transformation of the tube-side mass transfer coefficient derived in hollow fibre membrane contactors (HFMC) of different characteristic length scales (equivalent diameter and fibre length) has been studied for operation within the low Graetz range (*Gz*<10). For low *Gz* numbers, which corresponded to the region of gas phase controlled mass transfer, a proportional relationship between the experimentally determined mass transfer coefficient (*K*_*ov*_) and the Graetz number was identified. As the *Gz* number reduced below two, the experimental *Sh* number approached unity, which suggests that mass transfer is strongly dependent upon diffusion. However, within this diffusion controlled region of mass transfer, tube-side fluid velocity remained important. For *Gz* numbers coincident with where membrane resistance dominated mass transfer, *Sh* could be satisfactorily described by extension to the Lévêque solution, which can be ascribed to the constrained growth of the concentration boundary layer adjacent to the fibre wall. Importantly this study demonstrates that whilst mass transfer in the low Graetz range does not explicitly conform to either the Graetz problem or classical Lévêque solution, it is possible to transform the experimentally derived overall mass transfer coefficient (*K*_*ov*_) between characteristic length scales (*d*_*h*_ and *L*). This was corroborated by comparison of the empirical relationship determined in this study (*Sh*=0.36*Gz*) with previously published studies operated in the low *Gz* range. This analysis provides important insight for process design when slow tube-side flows, or low Schmidt numbers (coincident with gases) constrain operation of hollow fibre membrane contactors to the low *Gz* range. Specifically, this study evidences that estimation of the average Sherwood number using the Graetz problem would over predict mass transfer in the low Graetz range, by an order of magnitude, the practical implication for which is a gross underestimation of the membrane required to achieve a specific separation.

## Figures and Tables

**Fig. A1:**
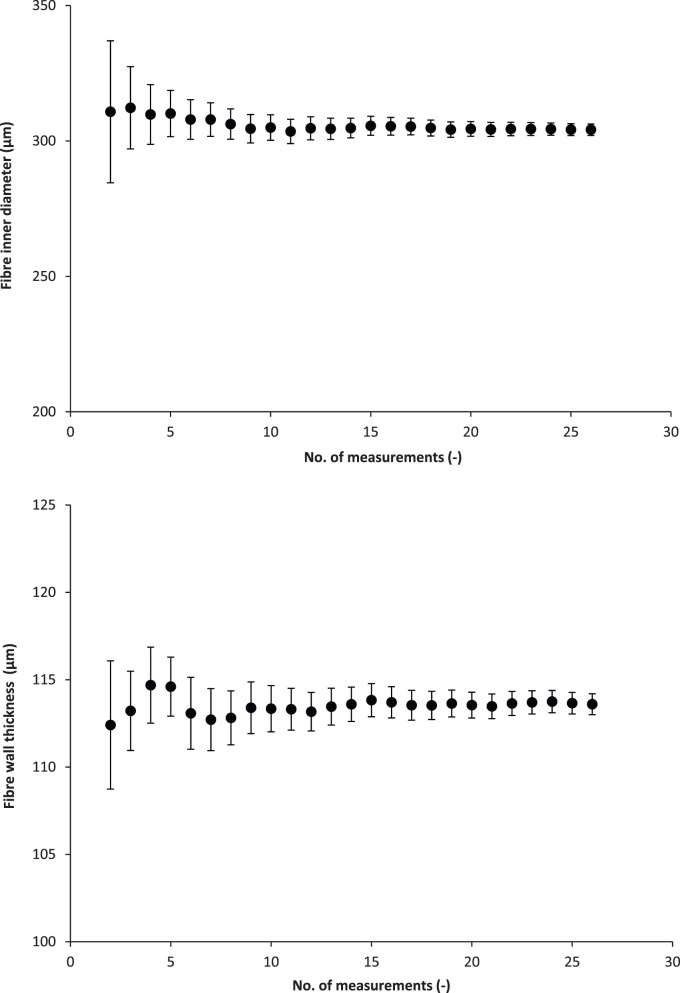
Both fibre inner diameter (lumen) and wall thickness was quantified for each PDMS hollow fibre lumen diameter evaluated (range 100–4800 µm). The 300 µm fibre is used here for illustration.

**Fig. A2:**
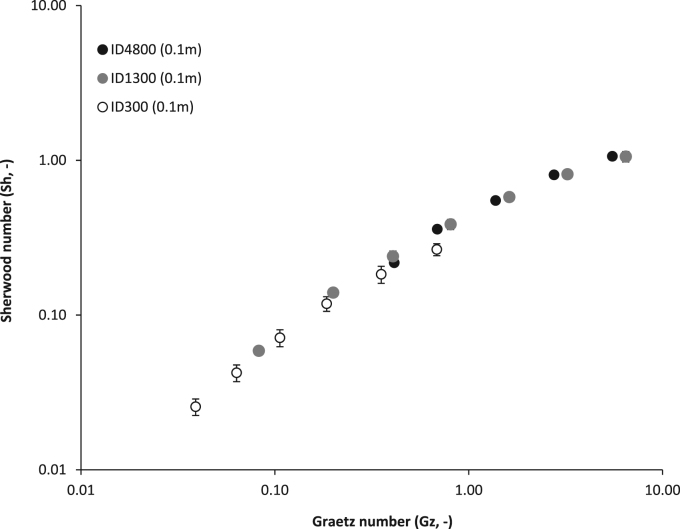
Illustration of data reproducibility. Each data point represents an average value of three experiments undertaken with three fibre of equal dimensions. Error bars represent the standard deviation which in all cases was below 10% of the Sherwood number reported.

**Table A1:** Data selected to provide mass transfer analysis from several applications.

Manufactured scale			Commercial	Commercial	Commercial
Application			O_2_ removal from water	Air humidification	O_2_ removal from water
Membrane type			PDMS	PDMS	Polypropylene^a^
			Dense, symmetric	Dense, symmetric	Microporous
Temperature		°C	22	22	22
Dynamic viscosity	µ	Pa s^−1^	0.954×10^−3^	1.847×10^−5^	0.954×10^−3^
Mass diffusivity	D	m^2^ s^−1^	1.97×10^−9^^b^	2.54×10^−5^	1.97×10^−9^^b^
Fluid density	ρ	kg m^−3^	998^c^	1.185	998^c^
Schmidt number	Sc	–	485	0.61	485
Hydraulic diameter	d_h_	m	190×10^−6^	190×10^−6^	210×10^−6^
Length	L	m	0.14	0.14	0.198
Diameter/length	d_h_/L	–	1.36×10^−3^	1.36×10^−3^	1.11×10^−3^
Fibre number	n	–	12,600	12,600	7047
Single lumen area^d^	a_SL_	m^2^	2.84×10^−8^	2.84×10^−8^	3.80×10^−8^
Total lumen area^d^	a_TL_	m^2^	3.57×10^−4^	3.57×10^−4^	2.56×10^−4^
Membrane area^e^	a_m_	m^2^	1.0	1.0	0.9

a Microporous.

b 20 °C.

c 21 °C.

d Based on lumen diameter.

e Available surface area for mass transfer.

**Fig. A3:**
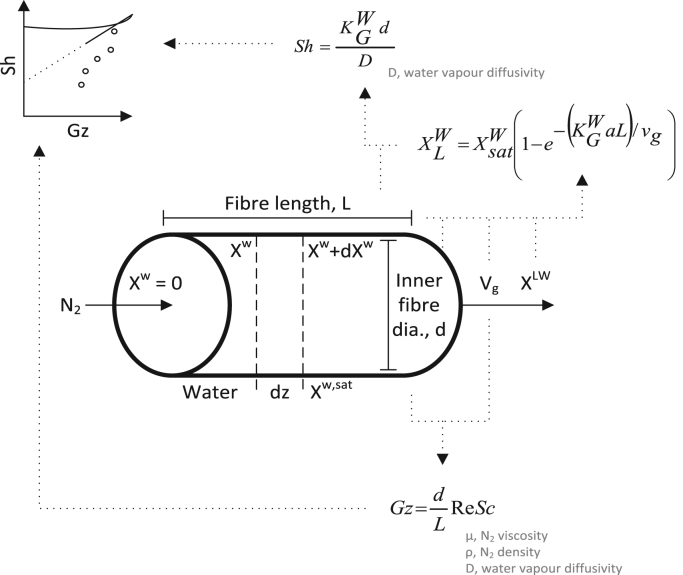
Summary of calculation procedure following measurement of gas-side (tube-side) data.

**Fig. A4:**
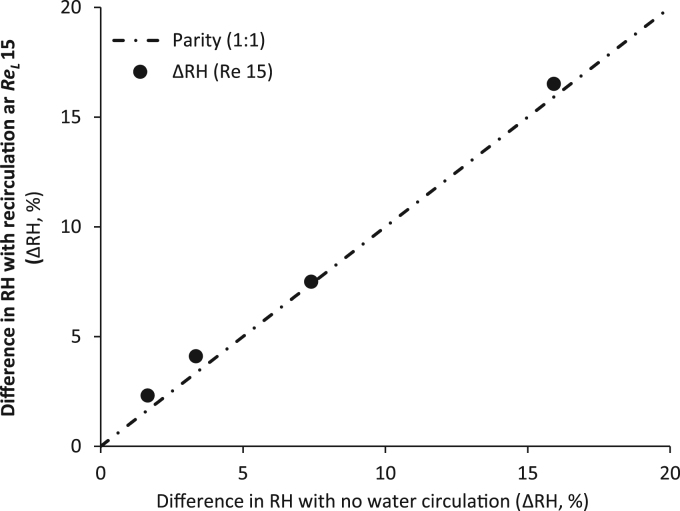
Comparison of outlet relative humidity data recorded when no water is recirculated compared to when water is recirculated at a Reynold’s number of 15.

**Table A2:** Membrane water vapour permeance.

Membrane material	Structural type	Wall thickness (cm)	Permeance (P/l)		Reference
			(cm s^−^^1^)	(GPU^h^)	
CTA^a^	TFC^b^	1.1×10^−4^	9×10^−3^	9000	[29]
SAC^c^	TFC^d^	5.0×10^−3^^d^	1.0×10^−2^	10,000	[39]
PDMS^e^	Symmetric	1.0×10^−2^	5.2×10^−4^^f^	520	[21]
PAN/PDMS^g^	TFC^b^	N/a	1.3×10^−2^	13,000	[42]

a Cellulose triacetate.

b Thin-film composite.

c Siloxane-amide copolymer.

d Estimated based on reported permeance and permeability.

e Polydimethlysiloxane.

f Estimated based on current fibre wall thickness in this study.

g PDMS-coated PAN composite.

h 1 GPU=1×10^−6^ cm^3^(STP) cm^2^ s^−1^ cm Hg^−1^.

**Table A3:** Module description used to determine the impact of liquid side hydrodynamics.

Module description		Units
*Fibre characteristics*		
Membrane material	PDMS	−
Wall thickness	100	µm
Lumen internal diameter	1800	µm

*Module characteristics*		
Shell volume	0.00184	m^3^
Shell area (for liquid flow)	0.0144	m^2^
No. of fibres	1	–
Total membrane area	0.00113	m^2^
Packing fraction	NA	m^−1^

*Module operation*		
Temperature difference (∆T_gas_)	0.07	°C
Temperature difference (∆T_gas/liquid_)	0.15	°C
Liquid side *Re*	0 and 15	–
Gas side *Re*	up to 800	–

**Table 1:** A non-exhaustive list of studies that have undertaken tube-side mass/heat transfer analysis.

Process	Solute	Valid range^a^	Sc	d_h_/L	Correlation	Ref.
Theoretical		Gz>20^b^	–	–	$\mathrm{Sh}=1.61{\mathrm{Gz}}^{1/3}$	[16]
Theoretical		Gz<10^c^	–	–	Sh=3.67	[16]
Theoretical		1st term	–	–	$\mathrm{Sh}=1.61{\mathrm{Gz}}^{1/3}-1.2$	[25]
Theoretical		1st and 2nd term	–	–	$\mathrm{Sh}=1.61{\mathrm{Gz}}^{1/3}-1.2-0.28057{\mathrm{Gz}}^{-1/3}$	[25]
Hollow-fibre membrane	Dissolved methane	Gz>10	671	1.95×10^−3^	$\mathrm{Sh}=1.61{\mathrm{Gz}}^{1/3}$	[24]
Hollow-fibre membrane	Dissolved oxygen	Re>5	507	4.2×10^−4^	$\mathrm{Sh}=1.61{\mathrm{Gz}}^{1/3}$	[36]
Hollow fibre membrane	Protein	N/s	–	–	$\mathrm{Sh}=1.5{\mathrm{Gz}}^{1/3}$	[8]
Hollow fibre membrane	Dissolved oxygen	Gz>4	476	0.71–2.4×10^−3^	$\mathrm{Sh}=1.61{\mathrm{Gz}}^{1/3}$	[41]
Tubular heat exchanger	Heat	Re>10	–	–	Sh=1.86Re1/3	[33]
Tubular heat exchanger/	Heat	Re<7	–	–	Sh=Re[0.5(ScdhL)2/3]^d^	[33]
Packed bed	Various (gas and liquid)	Pe<10	–	–	Sh=0.07Pe^e^	[12]
Hollow-fibre membrane	Humidity	Gz<2	0.55	1.25×10^−4^–4.8×10^−2^	Sh=0.36Gz	This study

a Evidenced by coherence of data to the relationship proposed.

b The limit generally stated.

c Not experimentally evidenced but often cited.

d Provided a gradient of 0.5 when considering Sc for Glycerol (~1040).

e Gradient estimated through data extrapolation.

**Table 2:** Estimated wall thickness and lumen diameter for each hollow fibre membrane lumen diameter studied (*n*=25).

	Wall thickness		Lumen diameter	
Stated	Measured	SE^a^	Measured	SE^a^
100	98.0	1.4	122.7	1.1
300	113.6	0.6	304.2	2.1
800	122.0	1.2	785.8	3
1300	138.0	2.0	1303.4	4.5
1800	115.5	2.7	1764.3	17.9
2800	113.2	2.5	2774.1	124.5
4800	128.9	4.7	4498.8	35.6
Average	118.5			
SD (%)	10.8			

a SE – Standard error.

## Appendix A Data reproducibility and literature data analysis

For each fibre, a dimensional characterisation was undertaken (wall thickness and lumen diameter). Measurements were taken until the standard error returned to a minimum. For illustration, the PDMS fibre with a manufacturer stated lumen inner diameter of 300 µm was characterised with a lumen diameter (tube inner diameter) and wall thickness of 304.2 µm and 113.6 µm respectively (Fig. A1).

Experimental data was produced in triplicate for each fibre diameter or fibre length studied, using three independent fibres of the same characteristic dimensions. The data contained within the main figures represent the average of this triplicate. To maintain the clarity of the data presented, error bars were not added. For illustration of data reproducibility, Fig. A2 presents Sherwood data from three fibres with fixed length (0.1 m) but different lumen diameters (300, 1300 and 4800 µm). The data presented is the average of a triplicate and the error bars represent the standard deviation of the triplicate, which in all cases was below 10% of the average Sherwood value reported.

Experimental data from the literature was also used to provide confirmation that the mass transfer behaviour exhibited within this study is more generally representative of mass transfer behaviour within the broader literature. Where available, the Sherwood data was extrapolated directly from the studies of interest [24], [41]. For several commercially available systems, the performance data was transformed by the authors into a Sherwood analysis using the assumptions presented in Table A1 and using the method presented in 
Fig. A3.

## Appendix B Extension of the Lévêque solution

For a hollow fibre membrane operated with fully developed, laminar flow within the lumen, through which a constant wall concentration is achieved, the Lévêque solution can be considered valid near the entrance. For considerable flow rates, the axial diffusion term can be neglected arriving at the Graetz problem. Newman [25] set out an to expansion of the Lévêque solution to small values of L, through inclusion of two variables, the Lévêque similarity variable (B1) and a dimensionless axial distance variable (B2), which are necessary for obtaining higher-order terms [25] (
Fig. A4).

(B1) $$\xi =\left(R-r\right){\left(4\left[V_{z}\right]/9D\left[\frac{d_{h}}{2}\right]L\right)}^{1/3}$$

(B2) $$\zeta =9\mathrm{DL}/4\left[V_{z}\right]{\left[\frac{d_{h}}{2}\right]}^{2}$$

From the dimensionless concentration $\theta =\left(c_{i}-c_{0}\right)/\left(c_{b}-c_{0}\right)$, the solution described as:

(B3) $$\frac{\delta^{2}\theta }{\delta \xi^{2}}+3\xi^{2}\frac{\delta \theta }{\delta \xi }-3\xi \zeta^{1/3}\frac{\delta \theta }{{\delta \zeta }^{1/3}}=\left(\frac{3}{2}{\xi^{3}\zeta }^{1/3}+\frac{\zeta^{1/3}}{1-\xi \zeta^{1/3}}\right)\frac{\delta \theta }{\delta \xi }-\frac{3}{2}\xi^{2}\zeta^{2/3}\frac{\delta \theta }{\delta \zeta^{1/3}}$$

with the boundary conditions θ=0atξ=0;θ=1atξ=∞;θ=1atζ=0. The solution can then be set-out as a power series in $\zeta^{1/3}$:

(B4) $$\theta =\theta_{0}\left(\xi \right)+\zeta^{1/3}\theta_{1}\left(\xi \right)+\zeta^{2/3}\theta_{2}\left(\xi \right)+0\left(\zeta \right)$$

in which $\theta_{0}\left(\xi \right)$ is the Lévêque solution. From this the ordinary differential equations for $\theta_{0},\theta_{1},\theta_{2}$ are:

(B5) $$\theta_{0^{"}}+3\xi^{2}\theta_{0^{\prime }}=0$$

(B6) $$\theta_{1^{"}}+3\xi^{2}\theta_{1^{\prime }}-3\xi \theta_{1}=\left(1+\frac{3}{2}\xi^{3}\right)\theta_{0^{\prime }}$$

(B7) $$\theta_{2^{"}}+3\xi^{2}\theta_{2^{\prime }}-6\xi \theta_{2}=\left(1+\frac{3}{2}\xi^{3}\right){\theta_{1^{\prime }}+\xi \theta }_{0^{\prime }}-\frac{3}{2}\xi^{2}\theta_{1}$$

with boundary conditions $\theta_{0}=\theta_{1}=\theta_{2}=0\;\mathrm{at}\;\xi =0,\theta_{0}=1,\theta_{1}=\theta_{2}=0\;\mathrm{at}\;\xi =\infty$. The solutions to the ordinary differential equations for $\theta_{0},\theta_{1},\theta_{2}$ are as follows:

(B8) $$\theta_{0}=\frac{1}{\Gamma \left(4/3\right)}\int_{0}^{\xi }e^{-x^{3}\mathrm{dx}}$$

(B9) $$\theta_{1}=-\frac{\xi^{2}e^{-\xi^{3}}}{10\Gamma \left(4/3\right)}-\frac{\left(3/5\right)}{\Gamma \left(4/3\right)}\xi \int_{\xi }^{\infty }e^{-x^{3}\mathrm{dx}}$$

(B10) $$\theta_{2}=-\frac{3/10}{\Gamma \left(4/3\right)}\xi^{2}\int^{\infty }e^{-x^{3}\mathrm{dx}}-\left(\frac{11}{210}+\frac{1}{14}\xi^{3}+\frac{3}{200}\xi^{6}\right)\frac{e^{-\xi^{3}}}{\Gamma \left(4/3\right)}+\frac{11}{630}\frac{\Gamma \left(5/3\right)}{{\left[\Gamma \left(4/3\right)\right]}^{3}}\int_{0}^{1}\frac{x^{1/3}}{{\left(1-x\right)}^{2/3}}e^{-\xi^{3}/\left(1-x\right)}\mathrm{dx}$$

The derivatives at the surface for $\theta_{0},\theta_{1},\theta_{2}$ are as follows:

(B11) $$\theta_{0^{\prime }}\left(0\right)=1/\Gamma \left(4/3\right)=1.11984652$$

(B12) $$\theta_{1^{\prime }}\left(0\right)=-0.6$$

(B13) $$\theta_{2^{\prime }}\left(0\right)=-\frac{11/140}{\Gamma \left(\frac{4}{3}\right)}{\left[\frac{\Gamma 5/3}{\Gamma 4/3}\right]}^{2}=-0.0899230$$

From this, the average Sherwood number relevant for the concentration difference at the inlet is:

(B14) $$\mathrm{Sh}=1.6151{\left(\frac{\mathrm{ReSc}}{L/d_{h}}\right)}^{1/3}-1.2-0.28057{\left(\frac{L/d_{h}}{\mathrm{ReSc}}\right)}^{1/3}+\ldots$$

## Appendix C Water vapour permeance of various membrane polymers

Literature data from previous studies evaluating water vapour transport in the gas phase (Table A2).

## Appendix D Impact of liquid velocity on mass transfer

Within this paper, we postulate that as the membrane fibre was immersed in water, the resistance associated with the liquid phase ($1/k_{L}^{W}S$) can be neglected and only the resistances associated with the membrane and gas phase need be considered. This is analogous to observations of previous authors [22]. To verify this assumption, we undertook tests on a single fibre exposed to water fluid flow (Table A3) and compared the data to that produced without fluid recirculation (Fig. A4). The arising data confirmed the negligible impact of fluid flow on mass transfer.
